# Breast Cancer Staging: Is TNM Ready to Evolve?

**DOI:** 10.1200/JGO.17.00004

**Published:** 2017-08-28

**Authors:** Fernando Cadiz, Juan G. Gormaz, Mauricio Burotto

**Affiliations:** **Fernando Cadiz**, **Juan G. Gormaz**, and **Mauricio Burotto**, Clínica Alemana de Santiago; **Mauricio Burotto**, Universidad del Desarrollo, Santiago, Chile.

The development of the TNM classification of malignant tumors has provided oncology with an invaluable tool for treatment decision making on the basis of cancer extent and spread. The TNM system is also essential for reliable outcome comparison of different interventions and accurate follow-up statistics. Therefore, it is currently the structural framework for prognosis description and determining the likelihood of successful outcomes for different treatment options indicated for every cancer stage. Beyond clinical practice, TNM has been a fundamental taxonomy and used for research purposes. However, during the previous two decades, oncology knowledge has increased exponentially, while, in our opinion, the TNM system update has evolved at a more conservative rate.

Cancer classification had its first hallmark in London, with the publication in 1932 of an article by Cuthbert E. Dukes, pathologist to St Mark’s hospital, entitled “The Classification of Cancer of the Rectum.”^1^ However, it was not until 1953 that the first TNM classification was finally published, by Dr Pierre Denoix.^2^ In 1958, the Union for International Cancer Control (UICC) produced the first international TNM recommendation for breast and larynx cancers. Between 1960 and 1967, the UICC published TNM classification proposals for 23 solid tumors, and the first TNM official version was edited in 1968.^3^ It is likely that in the early TNM classification years, designers did not envision the expansion and applicability of the system as it is used today, when most new clinical oncology knowledge cannot be separated from this staging system. Possibly the most outstanding characteristic of the TNM system is its broad standardization, mostly applicable, without differences, in almost every hospital worldwide. This has allowed epidemiologic comparisons from different regions, regardless of country development. Another key aspect of TNM is its simplicity for both use and interpretation. Staging starts with a simple clinical evaluation. In fact, this approach is the historic starting point at which clinicians estimate how localized or extended a tumor is.^4^

During the last decades, imaging and diagnostic improvements have resulted in migrations of the original TNM classifications. Small burden of disease in advanced-stage cancers (previously unrecognizable or underestimated in the standard TNM staging system) are now more precisely defined, causing the so-called stage migration, a phenomenon related to survival rate changes reported for similar cohorts treated in different decades and attributed to the use of new diagnostic procedures, which began to be described in the early 1980s.^5^ Therefore, in many cases, the TNM system may be delivering incomplete or less valuable information than it should, making it difficult or essentially incorrect to perform historical comparisons between patient cohorts. These limitations beg the question: Is this the time to review how we are staging and classifying tumors? If yes, will it be necessary to include certain current molecular knowledge in the taxonomy?

Although the TNM system has been the cornerstone of cancer classification for decades, current advances, mainly represented by molecular interrogation of different tumors, are becoming more relevant and are being used in addition to traditional staging in the initial patient approach, especially in colorectal, lung, and breast cancers. Virtually identical tumors in TNM classification and histologic type tend to show large differences in prognosis and high variability in treatment responses. For example, in localized or metastatic breast cancer, tumor biology, including hormone sensitivity, *HER2* status, cellular differentiation, and proliferation index, is fundamental to decision making and treatment planning.^6^ These variables also provide prognostic information regarding average overall survival and tumor recurrence, both for patients and doctors.^4-9^ Moreover, current guidelines, including those of ASCO, the National Comprehensive Cancer Network, and the St Gallen Consensus Conference, account for these factors in their recommendations, despite their absence from the TNM system. For example, the St Gallen Consensus Conference recommendations have already incorporated biologic subtypes in treatment recommendations in prognostic breast cancer classifications. Thus, there is a need to integrate new variables to staging classification in clinical practice, mobilizing the interest of organizations such as the Breast Cancer Task Force, which proposed the incorporation of tumor biology into the TNM staging system.^10,11^ Therefore, it seems to be reasonable to ask, Why is this not incorporated in the UICC standards?

One of the major arguments against expanding the current system with the aforementioned variables is that current cancer delineating and treatment methods are highly variable, depending on the economic development of respective countries. For example, despite a consensus about the importance of *HER2* as a predictor of a better outcome in localized and advanced disease,^4^ trastuzumab, an anti-HER2 monoclonal antibody, is not accessible to low-income patients with breast cancer in many countries. Therefore, the potential exclusion of a large number of patients worldwide must also be considered in any potential redesign of the TNM staging for the molecular era. Another reason that weighs against changing TNM as we know it, beyond its intrinsic success in generalizability and applicability, is that an inertia exists that favors maintaining a system that has worked well for decades, rather than innovation. On one hand, although there is no longer any doubt regarding the addition of biomarkers for improving outcome prediction,^12-14^ it is difficult to reach consensus about which should be included for a global standardization. On the other hand, because most survival analysis has been based on TNM staging, to modify this system is a major challenge in terms of reconciling traditional approaches with the new proposals. Finally, it cannot be denied that given the logistical difficulty of establishing these changes, the huge endeavor required, not only for related clinicians but also for all stakeholders in breast cancer treatment, plays against modifying TNM.

In conclusion, although precision in staging and prognosis of our patients has to be universally applicable, standardized, and as simple as possible, the current advances in breast cancer diagnoses and treatment make it necessary to incorporate molecular knowledge to TNM staging. However, TNM system modifications must be progressive and not disruptive, taking into consideration the cancer realities (eg, surgery, pathology, medical oncology) of developed and underdeveloped countries and the timings of breast cancer stakeholders worldwide. Therefore, new biomarker information must be incorporated gradually in a complementary manner that gives clear predictive information or can improve prognostic information compared with the actual TNM staging system.

## References

[1] Dukes CE (1932). The
classification of cancer of the rectum. J
Pathol
Bacteriol.

[2] Denoix PF (1953). Nomenclature
and classification of cancers based on an atlas [in
Spanish]. Acta Unio Int Contra
Cancrum.

[3] Greene FL, Sobin LH (2008). The
staging of cancer: A retrospective and prospective
appraisal. CA Cancer J
Clin.

[4] Sobin LH (2003). TNM:
Evolution and relation to other prognostic
factors. Semin Surg
Oncol.

[5] Feinstein AR, Sosin DM, Wells CK (1985). The
Will Rogers phenomenon. Stage migration and new diagnostic techniques as a
source of misleading statistics for survival in
cancer. N Engl J
Med.

[6] Loibl S, Gianni L (2017). HER2-positive
breast
cancer. Lancet.

[7] Harigopal M, Barlow WE, Tedeschi G (2010). Multiplexed assessment
of the Southwest Oncology Group-directed Intergroup Breast Cancer Trial
S9313 by AQUA shows that both high and low levels of HER2 are associated
with poor outcome. Am J
Pathol.

[8] Ross JS (2009). Multigene
classifiers, prognostic factors, and predictors of breast cancer clinical
outcome. Adv Anat
Pathol.

[9] Wang Y, Klijn JG, Zhang Y (2005). Gene-expression profiles
to predict distant metastasis of lymph-node-negative primary breast
cancer. Lancet.

[10] Edge SB, Byrd DR, Compton CC (2010). AJCC Cancer
Staging Manual.

[11] Carlson RW, Allred DC, Anderson BO (2009). Breast cancer. Clinical
practice guidelines in oncology. J Natl Compr
Canc
Netw.

[12] Yi M, Mittendorf EA, Cormier JN (2011). Novel staging system for
predicting disease-specific survival in patients with breast cancer treated
with surgery as the first intervention: Time to modify the current American
Joint Committee on Cancer staging system. J
Clin
Oncol.

[13] Ferguson NL, Bell J, Heidel R (2013). Prognostic value of
breast cancer subtypes, Ki-67 proliferation index, age, and pathologic tumor
characteristics on breast cancer survival in Caucasian
women. Breast
J.

[14] Goldhirsch A, Wood WC, Coates AS (2011). Strategies for subtypes:
Dealing with the diversity of breast cancer—Highlights of the St.
Gallen International Expert Consensus on the Primary Therapy of Early Breast
Cancer 2011. Ann
Oncol.

